# Vitamin D and the RNA transcriptome: more than mRNA regulation

**DOI:** 10.3389/fphys.2014.00181

**Published:** 2014-05-14

**Authors:** Moray J. Campbell

**Affiliations:** Department of Pharmacology and Therapeutics, Roswell Park Cancer InstituteBuffalo, NY, USA

**Keywords:** VDR, microRNA, transcriptome, epigenetic, microarray

## Abstract

The GRCh37.p13 primary assembly of the human genome contains 20805 protein coding mRNA, and 37147 non-protein coding genes and pseudogenes that as a result of RNA processing and editing generate 196501 gene transcripts. Given the size and diversity of the human transcriptome, it is timely to revisit what is known of VDR function in the regulation and targeting of transcription. Early transcriptomic studies using microarray approaches focused on the protein coding mRNA that were regulated by the VDR, usually following treatment with ligand. These studies quickly established the approximate size, and surprising diversity of the VDR transcriptome, revealing it to be highly heterogenous and cell type and time dependent. With the discovery of microRNA, investigators also considered VDR regulation of these non-protein coding RNA. Again, cell and time dependency has emerged. Attempts to integrate mRNA and miRNA regulation patterns are beginning to reveal patterns of co-regulation and interaction that allow for greater control of mRNA expression, and the capacity to govern more complex cellular events. As the awareness of the diversity of non-coding RNA increases, it is increasingly likely it will be revealed that VDR actions are mediated through these molecules also. Key knowledge gaps remain over the VDR transcriptome. The causes for the cell and type dependent transcriptional heterogenetiy remain enigmatic. ChIP-Seq approaches have confirmed that VDR binding choices differ very significantly by cell type, but as yet the underlying causes distilling VDR binding choices are unclear. Similarly, it is clear that many of the VDR binding sites are non-canonical in nature but again the mechanisms underlying these interactions are unclear. Finally, although alternative splicing is clearly a very significant process in cellular transcriptional control, the lack of RNA-Seq data centered on VDR function are currently limiting the global assessment of the VDR transcriptome. VDR focused research that complements publically available data (e.g., ENCODE Birney et al., 2007; Birney, 2012), TCGA (Strausberg et al., 2002), GTEx (Consortium, 2013) will enable these questions to be addressed through large-scale data integration efforts.

## The transcriptional landscape of the human genome

An appreciation of the diversity of transcription across the human genome has undergone a rapid expansion in recent years, in large part thanks to the efforts of integrative genomic approaches such as those of ENCODE consortium (Birney, 2012; Maher, 2012; Stamatoyannopoulos, 2012; Rosenbloom et al., 2013). From these studies it has become apparent that there is considerable variation and diversity in; the distribution of transcription factor binding across the human genome; the interplay between transcription factors and different co-regulating partners; the extent of the genome that is transcribed; the number and functionally different RNA-based molecules that are transcribed, the impact of mechanisms that process and edit RNA molecules that generate even greater diversity of gene expression.

In this context it is timely to review the functions of the vitamin D receptor (VDR/NR1I1) (Pike et al., 1980; Baker et al., 1988; Carlberg and Campbell, 2013), and consider how its actions contribute to this diversity of transcriptional and post-transcriptional events.

## The VDR acts in multimeric protein complexes to regulate transcription

The VDR, like many other members of the nuclear receptor superfamily are relatively well-understood transcription factors. Their actions have been dissected and modeled, and have generated the concept of cyclical gene regulation in which transcription factors oscillate between on and off states (Metivier et al., 2003; Reid et al., 2003; Kim et al., 2005; Vaisanen et al., 2005; Carroll et al., 2006; Meyer et al., 2006; Saramaki et al., 2006; Yang et al., 2006; Zella et al., 2006; Malinen et al., 2008; Seo et al., 2007).

A direct consequence of VDR genomic interactions and gene regulation is the control of the epigenetic states at receptor binding regions, and more broadly across target gene loci. Epigenetic events play a central role for transcriptional complexes and the various components in these multimeric complexes are able to initiate, sustain, and finally terminate transcription (Dobrzynski and Bruggeman, 2009). For example, different histone modifications can control the rate and magnitude of transcription (reviewed in Goldberg et al., 2007). These events are intertwined with levels of CpG methylation (Kangaspeska et al., 2008; Metivier et al., 2008; Le May et al., 2010). Thus the histone modifications regulated by VDR actions, and other epigenetic events including DNA methylation processes, combine during transcription to generate highly flexible chromatin states that are either transcriptionally receptive and resistant (Mohn and Schubeler, 2009). That is, the specific transcriptional potential of a gene is flexibly controlled by the combination of epigenetic events. These events are varied in space across the genomic loci, and in time through the course of the transcriptional cycle.

The diversity of histone modifications, and their association with different DNA functions formed the basis for the histone code hypothesis. This concept, first proposed in 1993, held that these modifications were governed in a coordinated manner and formed a code that mirrored the underlying DNA code to convey heritable information on transcription and expression (Turner, 1993). Given the rapid expansion of the understanding in the number of histone modifications, their genomic distribution and their combinatorial manner, it is only relatively recently that the true diversity of the range of histone states, and their functional outcomes, has become apparent (Goldberg et al., 2007). The strongest evidence that histone modifications at the level of meta-chromatin architecture form a stable and heritable “histone code,” is perhaps seen with X chromosome inactivation (reviewed in Turner, 1998). The extent to which similar processes operate to govern the activity of micro-chromatin contexts at gene promoter regions, is an area of debate (Jenuwein and Allis, 2001; Turner, 2002).

The regulation of transcription and the patterns of mRNA expression have been related to the expression of these histone modifications through a wide range of correlative and functional studies. For example, histone H3 lysine 4 tri-methylation (H3K4me3) is found in the promoter regions of actively transcribed genes. This mark is mutually exclusive with H3K9me, which instead is associated with transcriptionally silent promoter regions. Acetylation of H3K9 is found along with methylation of H3K4 at active promoter regions. Similarly, H3K27 can be either acetylated or methylated, with acetylation associated with active gene transcription and methylation associated with gene silencing.

**Figure 1:**
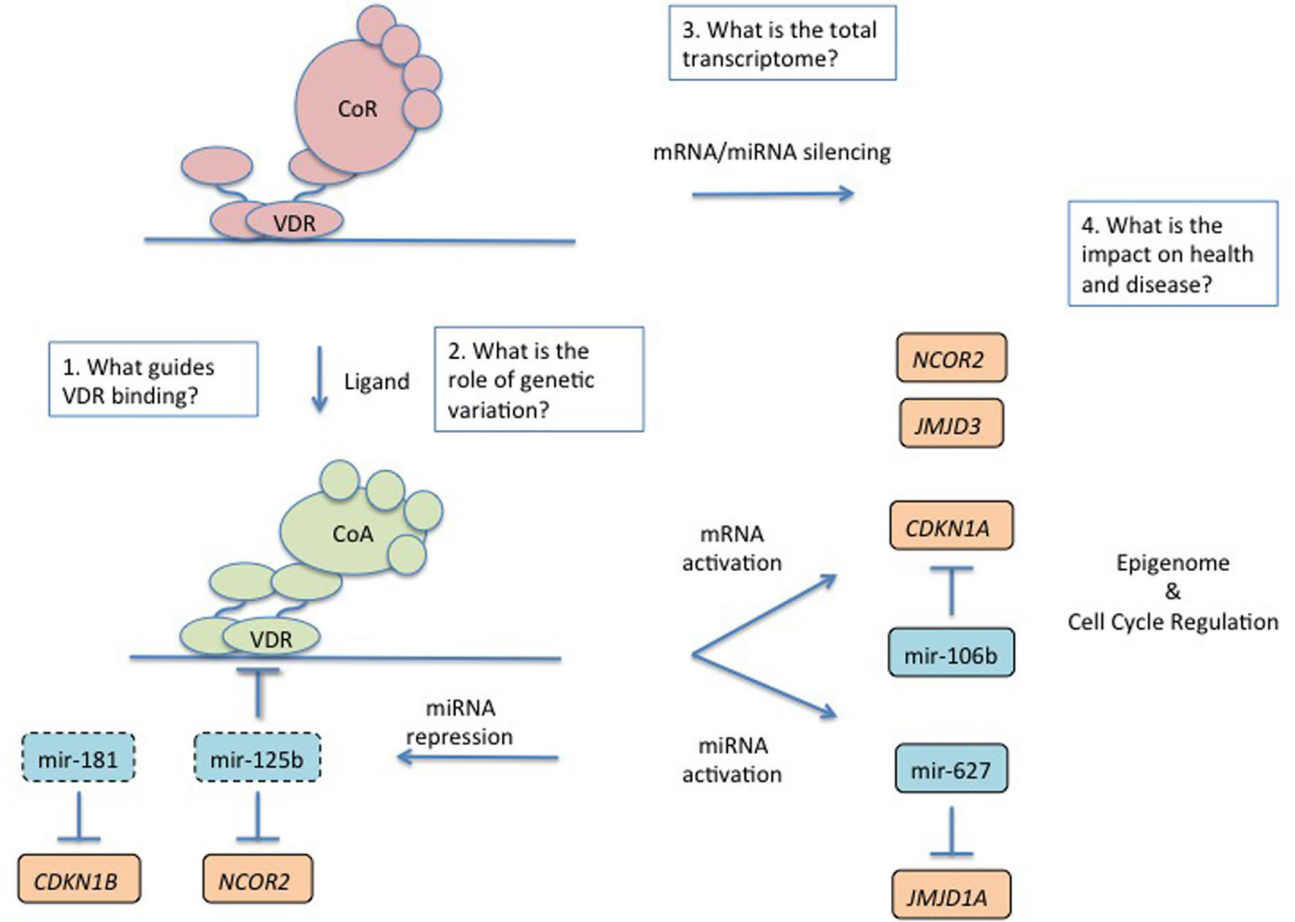
**An overview of regulation and impact of the transcriptome by the nuclear VDR and the future challenges**. The VDR toggles between a gene repressive and activating complex depending on the presence of ligand. Ligand activated gene regulated scenarios are most well understood. In the presense of ligand the VDR complex is associated with both mRNA and miRNA activation and there is clear evidence for co-regulation of these transcriptomes to tightly control final protein coding gene expression and phenotype. Ligand activated VDR also represses a number of miRNA for example including those that control expression of the VDR and components of the repressive complex. The major phenotypes associated with VDR control include cell cycle regulation and an emerging theme is the control of the epigenome. Four major questions remain in understanding VDR transcription. (1) What guides where the VDR binds in the genome? (2) What role does genetic variation in VDR binding sites play in changing the VDR transriptome? (3) What is the total VDR transcriptome? (4) What is the combined effect of the VDR transcriptome on health and disease and how can this be monitored and exploited?

In many experimental cases it has been established that VDR activation by natural ligand 1α,25-dihydroxyvitamin D3 (1α,25(OH)_2_D_3_) or synthetic analogs, can lead to a highly dynamic exchange of co-factors by releasing co-repressors and inducing a receptor conformation that attracts binding of co-activator proteins (Figure 1). This exchange of associations induces a more relaxed, or open, chromatin status and the recruitment of linking factors and subsequently the basal transcriptional machinery. However, this is not an indefinite signal and the ligand, is rapidly metabolized. Also the VDR itself is limited in function by proteasome-mediated receptor degradation (Peleg and Nguyen, 2010). In the absence of ligand, some basal level of receptor remains in the nucleus associated with co-repressor complex and leads to silencing of transcription (Malinen et al., 2008; Saramaki et al., 2009; Thorne et al., 2011; Doig et al., 2013). The sequencing, or choreography of these actions, give rise to the periodicity of transcriptional activation and pulsatile mRNA and protein accumulation and reflect intrinsic control mechanisms required to tightly regulate the expression of important signaling molecules. At closer resolution, for example on shorter time-scales, the patterns of regulation show some degree of coordinated pulsatile regulation, and probably reflect aspects of specific VDR binding sites impacting gene regulation for example through emerged to support chromatin looping within the same VDR target gene loci (Vaisanen et al., 2005; Saramaki et al., 2006, 2009).

Whilst these scenarios are relatively well characterized for the positive regulation of gene expression, it is probably not a complete understanding as the distribution of VDR binding in the genome (Ramagopalan et al., 2010; Heikkinen et al., 2011; Meyer et al., 2012; Ding et al., 2013) and the patterns of associated gene regulation suggest that the VDR is actually associated broadly with gene activation and repression. The mechanisms that drive gene repression appear more diverse than gene activation and reflect differences in complex formation, and the choreography of binding.

The patterns of protein-protein interaction identified for the VDR allude to both positive and negative gene regulation (Table 1). The VDR commonly forms a heterodimer with RXRα (Quack and Carlberg, 2000). However, the identification of partners that interact in the same complex supports a broad role for the VDR complex to regulate other signal transduction events and RNA processing activities.

**Table 1:** **Proteins known to interact with the VDR**.

**Interacting protein**	**Function**	**Detection method**	**Interaction**	**Publication**
Retinoid X receptor alpha (RXRα)	Transcription factor	Electron microscopy	Direct interaction	Orlov et al., 2012
Retinoid X receptor beta (RXRβ)	Transcription factor	Two hybrid	Physical association	Wang et al., 2011a
E1A-associated protein p300 (CBP/p300)	Histone acetyltransferase	Two hybrid	Physical association	Wang et al., 2011a
Mediator complex subunit (MED1)	Mediator complex that binds basal transcriptional machinery and drives transcriptional initiation	Pull down	Physical association	Yuan et al., 1998
Nuclear receptor coactivator 6 (NCOA6)	Transcriptional coactivator of multiple nuclear receptors and other transcription factors	Two hybrid	Physical association	Mahajan and Samuels, 2000
CXXC-type zinc finger protein 5 (CXXC5)	Transcription factor co-regulator of WNT signaling	Two hybrid	Physical association	Wang et al., 2011a
Tumor Protein P53 (p53)	Tumor suppressor protein containing transcriptional activation, DNA binding, and oligomerization domains	Fluorescence microscopy	Co-localization	Stambolsky et al., 2010
Protein naked cuticle homolog 2 (NKD2)	Antagonist of WNT via degradation DVL	Two hybrid	Physical association	Wang et al., 2011a
SMAD family member 3 (SMAD3)	Transcriptional effector of TGFβ	Pull down	Physical association	Leong et al., 2001
SNW Domain containing 1(SNW1)	Co-activator function with known roles as a splicing factor	Two hybrid	Physical association and co-localization	Baudino et al., 1998; Zhang et al., 2003
SFRS protein kinase 1 (SRPK1)	Serine/arginine protein kinase specific for the SR (serine/arginine-rich domain) family of splicing factors	Protein kinase assay	Phosphorylation reaction	Varjosalo et al., 2013
Protein kinase C substrate 80 K-H (PRKCSH)	Substrate for protein kinase C	Two hybrid	Physical association	Wang et al., 2011a
Protein-tyrosine phosphatase H1(PTPN3)	Protein tyrosine phosphatase that regulate a variety of cellular processes	Pull down	Physical association	Zhi et al., 2011
Complement Factor H (CFH)	Regulator of complement activation (RCA) gene cluster and plays a role in the defense mechanism to microbial infections	Two hybrid	Physical association	Wang et al., 2011a
β-catenin	Dual function protein, regulating the coordination of cell–cell adhesion and gene transcription.	Co-localization	Functional interaction	Pálmer et al., 2001
Prolylcarboxypeptidase (Angiotensinase C) (PRCP)	A lysosomal prolylcarboxypeptidase, which cleaves C-terminal amino acids linked to proline	Two hybrid	Physical association	Wang et al., 2011a
Cyclin D3 (CCND3)	Cyclin associated with control of cell cycle and known co-factor for several nuclear receptods	Two hybrid	Physical association	Wang et al., 2011a
Hair growth associated (HR)	Transcriptional corepressor of multiple nuclear receptors	Pull down	Direct interaction	Hsieh et al., 2003
Nuclear corepressor 1 (NCOR1)	Transcriptional corepressor	Two hybrid	Physical association	Tagami et al., 1998
Nuclear corepressor 2 (NCOR2)	Transcriptional corepressor	Immunoprecipitation	Physical association	Kim et al., 2009
COP9 signalosome subunit 2 (COPS2)	Transcriptional corepressor and component of the ubiquitin conjugation pathway	Two hybrid	Physical association	Polly et al., 2000

*The INTACT database curated by EBI http://www.ebi.ac.uk/intact/ was interrogated for interactions with the VDR. Interactions of VDR with NCOR1, NCOR2, and COPS2 were curated from the literature*.

A number of proteins with transcriptional activator function have been identified in complex with VDR. For example, CBP/p300 is a transcriptional co-integrator with histone acetlyase activity (Wang et al., 2011a,b, 2013a) and is associated with the VDR. Other proteins such as SNW1/NCOA62 which has function as a transcriptional co-activator (Baudino et al., 1998), as well as other proteins that have more recently been characterized to have coativator function (CCND3) (Cenciarelli et al., 1999; Lazaro et al., 2002; Despouy et al., 2003; Sarruf et al., 2005). Similarly the corepressor HR is also identified by such protein-protein interactions (Hsieh et al., 2003).

Aside from these traditional roles to modulate transcriptional actions, there is evidence to support a wider range of actions for the VDR in the control of mRNA. The coactivator SNW1 also plays a role as a splicing factor. This latter function is also shared by another VDR interacting protein, namely SRPK1, which is a protein kinase that regulates the activity of various splicing factors (Hayes et al., 2006; Aubol et al., 2013). Other interactions allude to the cross-talk between the VDR and different signal transduction processes. For example, the VDR interacts with negative regulators of WNT signaling (NKD2) (Katoh and Katoh, 2007), substrates for PKC signaling (PRKCSH) (Gkika et al., 2004), p53 (Kommagani et al., 2006; Lambert et al., 2006; Maruyama et al., 2006; Saramaki et al., 2006; Ellison et al., 2008) and SMAD3 (Ding et al., 2013; Ito et al., 2013; Zerr et al., 2014). The VDR functionally interacts with a range of co-repressors such as NCOR1 (Saramaki et al., 2009; Doig et al., 2013), NCOR2/SMRT (Khanim et al., 2004; Gynther et al., 2011), TRIP15/ALIEN (Polly et al., 2000; Cui et al., 2009) and DREAM (Scsucova et al., 2005) but, interestingly, agnostic protein-protein interaction tools such as INTACT (Table 1) have not identified direct VDR co-repressor interactions. It is unclear why these proteins are not identified in such protein-protein screens, but may reflect an experimental artifact as a result of investigators using ligand stimulated VDR to capture interacting proteins.

### The diversity of VDR-protein interactions is reflected by the distributions of genomic binding sites

To identify VDR binding sites through the genome several groups have now applied ChIP-Seq approaches in different human cell types including immortalized lymphoblastoids (Ramagopalan et al., 2010), hepatic stellate cells (Ding et al., 2013) and cancer cell lines representing monocytic leukemia (Heikkinen et al., 2011) and colon cancer (Meyer et al., 2012). These studies also differed in the time of cell exposure to 1α,25 (OH)_2_D_3_ ranging from 40 min to 36 h identify binding sites, on the order of hundreds to several thousands of different binding sites depending on time of treatment with 1α25(OH)_2_D_3_ and cell background, with longer treatment time points tending to be associated with more binding sites. However, as yet there are not uniform standards for the analyses of NGS data, and therefore it is likely that different analytical approaches are influencing the number and significance of the VDR enriched peaks identified.

These differences in treatment and analytical approaches aside, these VDR binding data sets reveals that fewer than 20% of the VDR binding sites are in common between the different cell types. These finding perhaps offers strong support for the concept that VDR transcription is extremely tailored in different cell types, presumably through interactions with either equal or more dominant co-factors that combine to determine its binding. Another important finding from these studies is that the canonical binding site for the VDR, termed the direct repeat (DR) spaced by 3 nucleotides (DR-3), which was identified by traditional biochemical approaches in candidate gene studies, appears to be the minority genomic element that directly binds the receptor. Fewer that 30% of genomic VDR binding sites contain a DR-3, although this number is increased following ligand treatment when there is increased enrichment for VDR binding to DR-3 elements (reviewed in Carlberg and Campbell, 2013). Nonetheless, a range of other genomic elements were enriched in VDR binding peaks suggesting that the VDR co-operates closely with other factors to associate with the genome, both in the absence and presence of ligand. Indeed, the study of Evans and co-workers in the hepatic stellate cells (Ding et al., 2013) and the work of Pike and co-workers in colon cancer cells (Meyer et al., 2012) both address this significant cross-talk of the VDR. In the case of the hepatic cells this is considered in the context of TGF® and in the case of colon cancer cells this is with TCF4, downstream of ®-catenin. Both of these studies therefore reflect the finding of VDR interactions with SMAD3 specifically, and more generally with regulators of WNT signaling (Table 1).

### The hunt for pioneer factors to explain the diversity of VDR function

Together these ChIP-Seq studies suggest that the VDR combines with other proteins in a network of interactions, quite likely in a cell type specific manner, to participate in diverse gene regulatory networks. It remains to be established how targeted or stochastic this is. The variation observed in both the type and position of binding sites for the VDR, depending on cell phenotype and disease state, suggests it is directed, and at least will establish a paradigm for hypothesis testing concerning what directs the VDR to bind and participate in gene transcription. The specificity of VDR signaling may arise due to integration with other perhaps more dominant transcription factors. Again, for other nuclear receptors (e.g., AR and ER) the concept has emerged that receptor binding is guided by the actions of more dominant so-called pioneer factors including the Forkhead (FKH) family members (Lupien et al., 2008; Serandour et al., 2011; Sahu et al., 2013). Efforts to define the major pioneer factors for the VDR have proved to be less consistent between the different VDR ChIP-Seq studies and may reflect the biology of the VDR which, given that it exists in the nucleus both in the presence and absence of ligand, potentially is a more interactive protein such that a single dominant pioneer factor is not so deterministic.

Another approach to identify the interacting partners of the VDR has been to examine the gene networks it regulates and to cluster genes by known regulating transcription factors. Novershtern et al. (2011) measured the transcriptome profiles of a large number of hematopoietic stem cells, multiple progenitor states and terminally differentiated cell types. They found distinct regulatory circuits in both stem cells and differentiated cells, which implicated dozens of new regulators in hematopoiesis. They identified 80 distinct modules of tightly co-expressed genes in the hematopoietic system. One of these modules is expressed in granulocytes and monocytes and includes genes encoding enzymes and cytokine receptors that are essential for inflammatory responses. Major players in this module are VDR together with the factors CEBPα and SPI1/PU.1. This indicates that VDR works together with this small set of transcription factors, in order to regulate granulocyte and monocyte differentiation. It is reasonable to anticipate that such modules exist in multiple cell types but are guided by the tissue specific expression of such factors.

### VDR regulation of the protein-coding transcriptome

Anti-proliferative effects of 1α,25(OH)_2_D_3_ have been demonstrated in a wide variety of cancer cell lines, including those from prostate, breast, and colon (Colston et al., 1982, 1989; Peehl et al., 1994; Campbell et al., 1997; Koike et al., 1997; Elstner et al., 1999; Welsh et al., 2002; Pálmer et al., 2003). Following on from these, VDR transcriptional studies were initially undertaken at the candidate level to identify processes by which the VDR mediated its cellular effects. These approaches identified of the gene encoding the 1α,25(OH)_2_D_3_ metabolizing enzyme *CYP24A1* (Dwivedi et al., 2000; Anderson et al., 2003) and *CDKN1A* (encodes p21*^(waf1/cip1)^*) (Schwaller et al., 1995) as VDR targets. Subsequently, with the emergence of differential expression and membrane array technology, workers applied these wider screening approaches to identify multiple genes regulated by the VDR. For example, Freedman and colleagues applied differential expression approaches in the context of 1α,25(OH)_2_D_3_ induced myeloid differentiation and identified a number of cyclin dependent kinase inhibitors including *CDKN1A* and undertook functional confirmation studies to suggest the importance of the regulation of these targets to trigger myeloid differentiation (Liu et al., 1996). Others undertook so-called focused array technology whereby cDNA probes for selected genes involved in key biological processes or disease states were arranged on macro scale membrane arrays. Such arrays contained anywhere from several hundred to several thousand probes, and so were not genome-wide in terms of coverage but rather were candidate arrays often focused around specific pathways or disease states such as cancer. Despite the limitations, these approaches yielded important information supporting the links between VDR action and the regulation of growth and signaling (Savli et al., 2002). Similarly, first generation arrays chips, for example from Affymetrix which contained 4500 probes (Akutsu et al., 2001), also enabled sufficient genomic coverage to begin to define specific regulated gene networks. This particular study from White (Akutsu et al., 2001) and co-workers identified 38 genes that were responsive to 1α,25(OH)_2_D_3_ exposure, which represented approximately 1% of the transcriptome studied, and included *GADD45A*. These earlier studies already suggested at the footprint of the VDR dependent transcriptome (reviewed in Rid et al., 2013). In many ways these studies highlighted the heterogeneity of VDR actions that was to be identified subsequently by ChIP-Seq studies. This heterogeneity may in part reflect experimental conditions with very different cell line differences, and genuine tissue-specific differences of co-factor expression that alter the amplitude and periodicity of VDR transcriptional actions.

Even within this diversity there is some consistency on a certain targets and the biological actions they relate to, including cell cycle regulation (Akutsu et al., 2001; Pálmer et al., 2003; Eelen et al., 2004; Wang et al., 2005). A common anti-proliferative VDR function is associated with arrest at G_0_/G_1_ of the cell cycle, coupled with up-regulation of a number of cell cycle inhibitors. Candidate promoter characterization studies have demonstrated a series of VDR binding sites in the promoter/enhancer region of *CDKN1A* (Liu et al., 1996; Saramaki et al., 2006). By contrast the regulation of the related CDKI p27^*(kip1)*^ is mechanistically enigmatic, and included translational regulation and enhanced mRNA translation, and attenuating degrading mechanisms (Hengst and Reed, 1996; Wang et al., 1996; Huang et al., 2004; Li et al., 2004). The up-regulation of p21*^(waf1/cip1)^* and p27*^(kip1)^* principally mediate G_1_ cell cycle arrest, but 1α,25(OH)_2_D_3_ has been shown to mediate a G_2_/M cell cycle arrest in a number of cancer cell lines via direct induction of *GADD45A* (Akutsu et al., 2001; Jiang et al., 2003; Khanim et al., 2004).

In the transition to genome wide understanding, workers applied more comprehensive array approaches to define VDR mRNA transcriptomes. For example, investigations of squamous cells (Lin et al., 2002; Wang et al., 2005) identified networks of genes that trigger the response to wounding, protease inhibition, secondary metabolite biosynthesis, cellular migration, and amine biosynthetic processes. Another approach has been to examine vitamin D sensitive and responsive isogenic cell pairs and undertake analyses to identify key networks that are critical for mediating antiproliferative sensitivity toward 1α25(OH)_2_D_3_. In this manner a critical role for TGF® signaling was again revealed, to associate with VDR antiproliferative sensitivity toward 1α25(OH)_2_D_3_ in breast cancer cell models (Towsend et al., 2006). Exploiting leukemia cell models with differential responsiveness toward 1α25(OH)_2_D_3_ triggered-differentiation (Tagliafico et al., 2006) identified that certain VDR transcriptional targets could distinguish the aggressiveness of the leukemia, again, focused around cell cycle and included *MS4A3* which can modulate the phosphorylation of CDK2 and therefore exert control over the cell cycle. This concept of VDR sensitive vs. resistant models was also exploited in prostate cancer to identify the critical VDR transcriptional targets that mediate antiproliferative sensitivity and again also identified cell cycle and signal transduction components including *GADD45A* and *MAPKAPK2* that were required to mediate the sensitivity of cells to 1α,25(OH)_2_D_3_. Furthermore, these studies examined the epigenetic basis for the transcriptionally inert state of these targets in resistant models (Rashid et al., 2001; Khanim et al., 2004).

Identification of the VDR-dependent transcriptome using microarray approaches is heavily dependent on a range of statistical and technical considerations (Do and Choi, 2006; Zhang et al., 2009) including hybridization variations and limitations, background effects, normalization procedures, the choice of statistical test to identify differentially expressed genes, which in turn relies on study design and the numbers of arrays and samples chosen for study. Many of these study components were only formally agreed upon with the establishment of the MIAME compliant protocols in 2001 (Brazma et al., 2001), and as these became accepted standards for journal publication these approaches became widespread through the biological community.

MIAME compliant array experiments are subsequently published in public archives, such as GEO (Barrett et al., 2005, 2013) and ArrayExpress at EMBL (Parkinson et al., 2007) and Stanford microarray database (Marinelli et al., 2008). These three repositories between them contain thousands of genome-wide microarray experiments, containing millions of individual microarrays. Mining these repositories reveals a range of experiments (not all published) where cells have been treated with 1α,25(OH)_2_D_3_ and other vitamin D compounds, and RNA effects studied between short time points (1–2 h) to several days (Table 2). Again, these studies have supported consistent themes in terms of the VDR-mediated control of cell cycle and signal transduction processes, the suppression of WNT and NF-κB, and the regulation of IGF1 signaling (Kovalenko et al., 2011), and integrated actions with TGF® signaling. A final area to emerge from these agnostic studies of the VDR transcriptome is the impact that 1α25(OH)_2_D_3_ exposure exerts on a range of chromatin remodeling components. Interestingly, NCOR2/SMRT appears to be a target of VDR signaling (Dunlop et al., 2004), and adds to the concept that VDR signaling is cyclical and based on the functions of various negative feedback loops. Similarly, KDM6B/JMJD3 is a histone H3 lysine demethylase and expression is induced by the activated VDR. In this manner, VDR action can feed-forward its own transcriptional program by promoting H3K9 acetylation and gene action (Pereira et al., 2011).

**Table 2:** **Publically available MIAME compliant microarray studies of VDR function**.

**Experimental title/design**	**GEO series accession number**	**Publication**
**PROTEIN CODING MRNA**		
Vitamin D effect on bronchial smooth muscle cells	GSE5145	Bosse et al., 2007
Genome-wide analysis of vitamin D receptor (VDR) target genes in THP-1 monocytic leucemia cells	GSE27270	Heikkinen et al., 2011
Transcriptional effects of 1,25 dihydroxi-vitamin D3 physiological and supra-physiological concentrations in breast cancer organotypic culture	GSE27220	
Analysis of vitamin D response element binding protein target genes reveals a role for vitamin D in osteoblast mTOR signaling	GSE22523	Lisse et al., 2011
Expression profiling of androgen receptor and vitamin D receptor mediated signaling in prostate cancer cells	GSE17461	Wang et al., 2011b
Understanding vitamin D resistance using expression microarrays	GSE9867	Costa et al., 2009
Effects of TX527, a hypocalcemic vitamin D analog on human activated T lymphocytes	GSE23984	Baeke et al., 2011
Transcriptome profiling of genes regulated by RXR and its partners in monocyte-derived dendritic cells	GSE23073	Szeles et al., 2010
**NON-PROTEIN CODING RNA**		
MicroRNA-22 upregulation by vitamin D mediates its protective action against colon cancer.	GSE34564	Alvarez-Diaz et al., 2012
miRNA profiling of androgen receptor and vitamin D receptor mediated signaling in prostate cancer cells	GSE23814	Wang et al., 2013b
Identification of miRNAs regulated by vitamin D within primary human osteoblasts	GSE34144	
Vitamin D and microRNA expression	GSE20122	

Given the number of arrays available, it is now timely to consider meta-analyses across the arrays to reveal common themes; this forward compatibility is one the key benefits of MIAME compliance. Meta-integration of array data has been shown to be surprisingly revealing in a range of studies. For example at the larger scale various workers have integrated multiple microarray data to reveal underlying patterns in the context of disease classification (Shah et al., 2009; Engreitz et al., 2011) but can also be applied to consider that specific phenotypes (Martinez-Climent et al., 2010; Rantala et al., 2010; Lai et al., 2014). It is therefore timely for these data to mined, and integrated with related nuclear receptor actions or other transcription factors that appear to co-operate with the VDR, for example SMADs.

### VDR regulation of non-coding RNA species

The human genome project in many ways was a race to define the protein coding genes within the human genome. Bacterial artificial chromosomes (BAC) clones enabled relatively large pieces of DNA, upto 300 kb to be inserted for sequencing (Osoegawa et al., 2000). However, a key step in the initial alignment process was to leverage cDNA and EST libraries and therefore naturally steered workers to protein coding genes, and the significance and extent of non-protein coding RNA remained largely unexplored.

Although non-coding RNA forms were well described in terms of ribosomal function it was little understood beyond this. The interpretation of the human genome, and other large scale approaches to investigating chromosomal function (Consortium et al., 2007; Tress et al., 2007) all led to a growing awareness of the extent of non-coding RNA and at least suggested that their was an unknown. This uncertainty has been reflected in the debate within the biological community over the extent and roles of so-called Junk DNA (Kapranov and St Laurent, 2012; Doolittle, 2013). As a result researchers have considered roles for non-coding RNA in the regulation of cell function and have begun to examine the interplay between the at least 20 different types of different non-coding RNA (reviewed in Ling et al., 2013). Many of these RNA species are gene regulatory RNA and include microRNA (miRNA), long non-coding RNA (long ncRNA), whereas others are involved in the post-transcriptional modification of RNA for example small nucleolar RNA (snoRNA).

Workers have now principally examined miRNA regulation by the VDR and evidence has emerged to support a role for the VDR to control regulation. For example Studzinski and co-workers revisited the mechanistically enigmatic VDR-mediated control p27*^(kip1)^*. They elegantly demonstrated a role for VDR to down-regulate miR181a, which when left unchecked degrades p27*^(kip1)^*(Wang et al., 2009) (Figure 1). Thus, indirectly, VDR activation elevates expression of p27*^(kip1)^*, initiates cell cycle arrest and commits cells toward differentiation. These studies illuminated the earlier ones that suggested that p27*^(kip1)^* protein levels appeared to be regulated by a range of post-transcriptional mechanisms, such as enhanced mRNA translation, and attenuating degradative mechanisms (Hengst and Reed, 1996; Huang et al., 2004; Li et al., 2004). Similar integration of miRNA and mRNA was revealed to control the regulation of *CDKN1A*. Dynamic patterns of *CDKN1A* mRNA accumulation have been observed in various cell systems (Thorne et al., 2011). This is in part explained by the epigenetic state of different VDR binding sites on the *CDKN1A* promoter. However, VDR-dependent co-regulation of miR-106b also appears to modulate the precise timing of *CDKN1A* accumulation and expression of p21*^(waf1/cip1)^* in a feed-forward loop and determine the final extent of the cell cycle arrest. 1α,25(OH)_2_D_3_ regulates the DNA helicase *MCM7* (Khanim et al., 2004) that encodes the miR-106b, in intron 13 of the *MCM7* gene, and together these co-regulation processes control p21*^(waf1/cip1)^* through the balance of MCM7 and *CDKN1A* (Saramaki et al., 2006; Ivanovska et al., 2008) (Figure 1).

MicroRNA (miRNA) contribute negative regulatory aspects to normal gene regulation, for example as part of feed-forward loop motifs (Mangan and Alon, 2003; Mangan et al., 2006). The co-regulation of mRNA and miRNA in motifs that included feed forward structures appears quite common (Song and Wang, 2008; Gatfield et al., 2009; Ribas et al., 2009; Sun et al., 2009; Wang et al., 2009). Other established miRNA targets of the VDR include miR-627 (Padi et al., 2013) that in turn targets *JMJD1A* (another histone H3 lysine demethylase) miR-98 (Ting et al., 2013) and let-7a-2 (Guan et al., 2013). However, one of the more explored relationships between VDR and miRNA is the relationship between VDR and miR-125b. MiR-125b inhibits VDR (Mohri et al., 2009; Zhang et al., 2011) and in turn VDR can down-regulate miR-125b (Iosue et al., 2013; Zhou et al., 2014), and the other targets of miR-125b include NCOR2/SMRT (Yang et al., 2012) (itself a VDR target gene) suggesting multiple levels of co-regulation and interdependent relationship between the VDR, and the mRNA and miRNA transcriptomes, and the epigenome. Finally, it is interesting to note that altered levels of miRNA are associated with cancer states and progression risks and indeed miR-125b is associated with aggressive prostate cancer (Shi et al., 2011; Amir et al., 2013; Singh et al., 2014).

Beyond these candidate studies, a number of investigators have undertaken miRNA microarray analyses (Table 2) and these approaches have identified various networks, including the control of lipid metabolism and PPAR^©^ function (Wang et al., 2013b). It is likely that with the increased application of array approaches and next gen sequencing approaches will identify the key networks downstream of the VDR miRnome. This is unfortunately a more challenging research question owing to the many-to-many nature of miRNA; a given miRNA target many mRNA, and a given mRNA may have many miRNA targeting it. Thus, the computational challenges to resolve these relationships are not insignificant. Together these findings suggest that co-regulated miRNA may form an integral part of VDR signaling to control gene expression.

Of the other types of non-coding RNA, their regulation by VDR remains far less explored. Recently the group of Bickle have begun to dissect VDR regulation of lncRNA in keratinocytes and identified a number of target lncRNA and in doing so have raised the curtain on new avenues of exploration (Jiang and Bikle, 2014).

## Conclusions and future challenges for understanding the VDR transcriptome

### What are the proteins, or processes that guide where the VDR binds in the genome?

It is unclear what key pioneer factors will be identified and if the VDR is in a strong relationship with a specific family of pioneer factors, in the same that the AR is profoundly influenced by the Forkhead family members. Indeed, the precise pioneer factor may even be tissue specific as also revealed for the AR (Pihlajamaa et al., 2014). This may reflect the fact the ligand activation of the VDR is more associated with re-distribution of the VDR through the genome, rather than triggering movement into the nucleus (as in the case of the classic steroid hormone receptors).

The specific epigenetic niche that characterizes the VDR binding may also be revealing of where and why the VDR binds to the genome. These analyses will require agnostic integration of multiple genomic data sets, for example histone modifications, transcription factor binding, chromatin conformation and transcriptomic data and application of machine learning approaches to reveal the significance of the underlying patterns, VDR binding and transcriptional activity. Whilst the VDR was not included in the ENCODE project, judicious choice of a cell line model for these studies, most likely a Tier 1 cell line from ENCODE, will enable leverage of a considerable volume of cistromic and epigenomic data to be combined with *de novo* VDR ChIP-Seq data. In this manner the question of how TGF® and/or WNT signaling interacts directly or indirectly with VDR binding can be addressed relatively easily.

Another major knowledge gap in VDR understanding concerns the spatial relationships between VDR binding and the control of transcription. It is clear that chromatin looping processes can transiently bring distal regulatory regions into physical proximity to the proximal regions of a gene and lead to dynamic gene expression (Saramaki et al., 2009). To transition from examination of looping of this process at a single locus using established binding sites to the genome wide investigation is technically and statistically very challenging. Again, ENCODE analyses may be useful here, as Chromatin Interaction Analysis by Paired-End Tag Sequencing (ChIA-PET) data are available for specific cell lines; for example using RNA-PolII in K562 cells and therefore VDR ChIP-Seq in these cells would again be able to leverage this data to begin to understand how the VDR distributes and loops across the genome.

### What is the role of genetic variation in determining how and where the VDR binds?

Genetic variation exists throughout the genome and by definition is predominately in non-RNA coding regions. This realization has been the catalyst for examining how genetic variation impacts transcription factor binding and activity. Perhaps the most comprehensive integration of these concepts has been the development of the RegulomeDB tool (Boyle et al., 2012) which considers the impact of genetic variation on the function of all transcription factors analyzed by ENCODE. To date this question has not been seriously considered in terms of the VDR. A major hurdle to addressing this question is very large potential for Type 1 error owing to the large-scale data sets that need to be integrated, namely; all SNPs and those in linkeage disequilibrium that are significantly associated with disease in replicated studies and all binding sites for a given transcription factor against the backdrop of the number of SNPs for a given trait, the platform used for identification and the total number of SNPs in the human genome. In the context of the VDR specifically, this challenge is compounded by the fact that the majority of VDR binding sites through the genome do not contain a canonical DR3 type binding element and therefore a critical question will remain around what protein is the VDR interacting with in the genomic context and how is this influenced by genetic variation. This challenge is clearly intertwined with developing a comprehensive knowledge of how the VDR binds to the genome.

### What is the complete VDR transcriptome and how does it differ by tissue and by disease?

Surprisingly, no RNA-Seq data are yet available for the VDR. Therefore to capture all RNA regulated by the VDR will require RNA-Seq approaches applied to libraries that capture short and long RNA species. The ENCODE consortium have undertaken over 400 RNA-Seq experiments focused on different RNA species in multiple cell models, and this makes a compelling case for exploiting these data, especially in Tier 1 or Tier 2 cell lines. For example undertaking VDR ChIP-Seq and a limited number of RNA-Seq approaches in K562 cells has the potential to leverage a remarkable volume of data. Predictions from such integrative analyses could then be tested in other ENCODE resources, in RNA-Seq from multiple tissues in normal healthy donors, through the GTEx consortium, or through the vast numbers of microarrays that are publically available. For example, in the case of K562, which is a CML cell line, there are many large-scale microarray analyses of patients with CML to examine how the VDR transcriptome relates to disease state and drug response. A parallel outcome of investigating VDR function will be to address the role it plays in regulating splice variation as suggested by the interactions with proteins such as SNW1.

### How will this knowledge be exploited in personalized measures of VDR system in health and disease?

Many aspects of the relationships identified above can be interpreted by serum borne measurements, which are highly attractive owing to their ease of measurement. Serum levels of the pro-hormone, 25(OH) vitamin D3, are strongly correlated with the generation of the active hormone 1α,25(OH)_2_D_3_ and VDR function. For example, reduced serum levels of 25(OH) vitamin D3 levels are associated with increased risk of either cancer initiation and/or progression (Drake et al., 2010; Shanafelt et al., 2011). Therefore the serum level of 25(OH) vitamin D3can yield the “potential” of the VDR system to signal (Brader et al., 2014). This potential is impacted by the various cellular mechanisms outlined above. Of these, genetic variation that impacts VDR binding can obviously be measured in any cell in the body. The total transcriptome can be challenging to measure but perhaps small non-coding RNA represent a highly attractive marker of activity. Remarkably, miRNA are readily secreted into serum where they remain stable (El-Hefnawy et al., 2004; Goyal et al., 2006; Taylor and Gercel-Taylor, 2008; Valadi et al., 2007) and can be reliably extracted and measured (Chen et al., 2008; Liu et al., 2008; Mitchell et al., 2008; Rabinowits et al., 2009). Using serum-borne molecules as prognostic markers is highly attractive for several reasons. First, they can overcome the limitations of inaccurate sampling for example in the case of the presence of cancer within a tumor biopsy. Second, they can encapsulate the effects of heterotypic cell interactions, again, for example within the tumor microenvironment. Third, they form a non-invasive test procedure. From a biostatistical perspective, given there are fewer miRNA than protein coding mRNA, genome-wide coverage is more readily achieved and avoids the statistical penalties typically associated mRNA genome wide testing (Lussier et al., 2012).

This raises the very exciting possibility that generating integrated models of VDR binding, the impact of genetic variation, the tissue specific differences in the transcriptome and identifying miRNA contained within transcriptional circuits offers the opportunity of exploiting their serum expression levels of 25(OH) vitamin D3, genetic variation and miRNA expression will be able to be exploited to predict accurately the capacity of VDR function.

### Conflict of interest statement

The author declares that the research was conducted in the absence of any commercial or financial relationships that could be construed as a potential conflict of interest.
